# The Efficacy of Well-Being Therapy for Depression in Infertile Women

**DOI:** 10.22074/ijfs.2016.5087

**Published:** 2016-11-01

**Authors:** Majid Moeenizadeh, Haniyeh Zarif

**Affiliations:** Department of Psychology, Faculty of Education and Psychology, Ferdowsi University of Mashhad, Mashhad, Iran

**Keywords:** Infertility, Depression, Psychological Well-Being, Well-Being Therapy

## Abstract

**Background:**

Infertility is a major public health problem with physical, psychological and social dimensions. High prevalence of psychological problems has been reported in infertile women. The objective of this study was to examine the effectiveness of well- being therapy (WBT) for depression in infertile women who were referred to an infertility center in Mashhad, Iran.

**Materials and Methods:**

This preliminary trial was conducted at the Montasariya Infertility Center, Mashhad, Iran, between July and October 2011. A group of 22 infertile women were randomly assigned into experimental (n=11) and control groups (n=11). Patients were assessed with two self-rating inventories including the Psychological Well- being (PWB) and the Depression, Anxiety and Stress Scale-21 (DASS-21) before and after the interventions and the waiting-list period. WBT was performed in 8 to 10 sessions according to the published protocol.

**Results:**

Analysis of covariance (ANCOVA) showed a significant difference regarding the depression scores of experimental group between preand post-treatment as compared to control subjects.

**Conclusion:**

The results suggested the feasibility and clinical advantages of adding WBT to repertoire of the treatment techniques for depression in infertile women.

## Introduction

Infertility is a stressor that affects not only women but also their husbands. Infertility is more stressful and unacceptable for women, although the signs of depression in men/husbands have been indicated (1). In a study by Litt et al. (2), they have measured optimism, specific expectancies for fertilization success, coping strategies, and distress levels in people whose attempts were unsuccessful, approximately 8 weeks before the attempt. The results of this experimental study have showed that the distress returned just two weeks after notification of a negative pregnancy test.

In another recent study by Bleil et al. (3), they have examined the influence of optimism on infertility treatment among 198 women. The treatment results were categorized as successful and failed *in vitro* fertilization (IVF) treatment cycle. By the end of the 18-month study, the participants were divided into two groups of having delivered a baby and being pregnant due to cycle. At baseline, optimism and pessimism were also measured. They have indicated pessimism as a risk factor in failure of IVF treatment.

Lancastle and Boivin (4) have studied psychological variables on reproductive health on 97 women. Their primary outcomes were optimism, anxiety, and coping with problems three months before fertility treatment. The secondary outcomes were the biological response to treatment (e.g. estradiol level). Their findings have shown that optimism is the result of its shared variance with neuroticism. They have concluded that psychological variable were significant indicators of a single concealed structure. In another study conducted in Greece, 137 infertile women were evaluated to measure stress (infertility-related stress, anxiety, symptoms of depression, and mood states), character peculiarities (neuroticism, extraversion, optimism) and coping strategies using the Appraisal of Life Events (ALE). The finding of study confirmed that ALE had a satisfactory reliability and convergent validity (5).

Although one of the main problems of the couples is primary infertility worldwide, the rate of secondary infertility increases in the third world countries, in which the cost of treatment as well as social and cultural factors are considered as the important factors in restricting access to fertility treatment (6).

The global infertility prevalence rates are difficult to determine, but according to the report by World Health Organization (WHO), published at the end of 2012, one in every four couples in developing countries had been found to be affected by infertility. Marriage as a social phenomenon is negatively affected by infertility that leads to poor physical and mental health of individuals (7).

### Physical health and welfare

**Fig.1:**
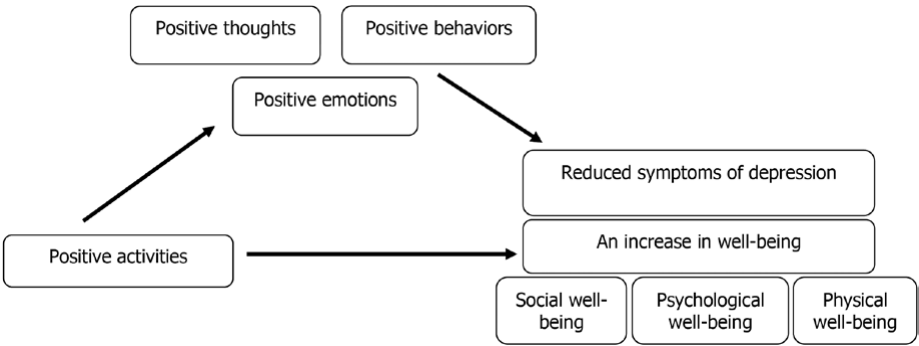
The effectiveness of positive psychology interventions in reducing depressive symptoms and increasing well-being.

Positive emotions are the key points in improving well-being. According to Layous et al. (8), when moderating variables, such as positive behavior, positive thoughts and positive emotions, are accompanied by with positive activities, they diminish depressive symptoms and improve well-being. Such results led to represent the following model (Fig .1) which points out the effectiveness of cognitive interventions in reducing depressive symptoms and increasing positive well-being in the clients.

It seems that there is an undeniable and clear relationship between positive emotions with physical health and well-being because illnesses are accompanied by pain and unhappiness. Disease is often recognized as an increasingly negative effect, while it may cause movement disorder which also decreases the positive effect and satisfaction (9).

Rayan and Frederick (10) have reported positive emotion of vivacity and vitality. According to their findings, mental vigor is the sign of organismic well-being that should be accompanied with both psychological and physical factors affecting the supply of energy to the individual.

### Well-being therapy

Well-being therapy (WBT) enhancing positive psychology intervention was developed by Fava et al. (11-17) in Italy and validated in a number of controlled trials. WBT is based on an educational model which is structured, directive, and problem-oriented to present problems and situations. The duration of each session is 45 to 60 minutes. The therapy is over a period of eight weeks. WBT includes technique of self-observation along with the use of a structured diary and interaction between patients and therapists. The therapy sessions are divided into three phases-initial, intermediate and final. The first two sessions (the initial phase) are for identifying incidences of well-being and applying the rules into situational context regardless of its short period (13). Patients are requested to maintain report in the form of a structured diary to explain the circumstances surrounding the episodes of well-being, rated on a scale of 0 to 100, with 0 indicating absence of well-being and 100 indicating the most intense well-being. In the 3rd to 5th sessions (the intermediate phase), patients are encouraged to recognize thoughts and beliefs that lead to early cutting off of well-being. In the 6th to 8th sessions (final phase), patients are assessed according to following Ryff 's six dimensions of psychological well-being (PWB) scales: i. Environmental mastery, ii. Personal growth, iii. Purpose in life, iv. Autonomy, v. Self-acceptance, and vi. Positive relations with others (18). Therefore, the objective of this study was to examine the effectiveness of WBT for depression in infertile women who were referred to an infertility center in Mashhad, Iran.

## Materials and Methods

In this preliminary trial, patients (n=22) were randomly assigned to an experimental group (n=11) and a control group (n=11) to find out the efficacy of WBT for depression in infertile women, with assessment before and after therapy.

### Samples

The sample consisted of infertile women (20 to 40 years old) who visited the Montasariya Infertility Center, Mashhad, Iran, between July and October 2011. Twenty-two infertile women suffering from depressive disorders as per Diagnostic and Statistical Manual of Mental Disorders, 4^th^ Edition, Text Revision (DSM-IV-TR) criteria were chosen for the study using Krejcie and Morgan (19) sample size table.

### Ethical consideration

We obtained the permission of the Montasariya Infertility Center to conduct this study. We also explained our aim to participants separately before they signed an informed consent, although we did not ask them to include their names in the questionnaire.

### Assessments

PWB and the Depression, Anxiety and Stress Scale-21 (DASS-21) were used by the researcher prior to the treatment. The first and second sessions were spent on administrating Persian version of DASS-21 including a quantitative measure of distress along with the three axes of depression, anxiety and stress (20) and PWB consisting of subjective, social and psychological dimensions as well as health-related behaviors (21). The subjects were reassessed with the DASS-21 and PWB after treatment (8 sessions) by the same researcher who had performed the previous evaluations and who was blind to the treatment assignment. According to Samani and Jokar (20), the reliability and validity of DASS-21 ranged from 0.74 to 0.81. They also showed that the test-retest reliability values for depression, anxiety and stress were 0.80, 0.76, and 0.77, respectively, while Cronbach's alpha values for depression, anxiety and stress were 0.81, 0.74, and 0.78, respectively. Furthermore, for the validity of this scale, the confirmatory factor analysis and principal components method were utilized. Kaiser-Meyer-Olkin (KMO) measure was equal to 0.9012 and the Bartlett’s test of sphericity (x2) was equal to 3092.93.

The second scale with 84 items was utilized to evaluate six dimensions of PWB as follows: i. Autonomy, ii. Environmental mastery, iii. Personal growth, iv. Positive relationships with others, v. Purpose in life, and vi. Self-acceptance. Each dimension was measured with 14 items. The original 20-item per scale version of the PWB was validated in a community-based sample of 321 men and women from multiple age groups (22). Analyses represented that each of the six scales had high levels of internal consistency with alpha coefficients ranging from 0.86 to 0.93. Test-retest reliability (over 6 weeks) was acceptable, ranging from 0.81 to 0.88 for the six scales. The scales also showed good construct validity with significant correlation with Bradburn’s Affect Balance Scale (correlation coefficients ranged from 0.25 to 0.62), Neugarten’s Life Satisfaction Index (LSI) (correlation coefficients ranged from 0.26 to 0.73, all P<0.001) and Rosenberg Self-Esteem Scale (RSES) (correlation coefficients ranged from 0.36 to 0.62, P<0.001). This scale was translated into Persian by this investigator. The reliability was calculated (n=30 students) and Chronbach coefficient alpha ranged from 0.88 to 0.96.

### Procedure

In this 8-week program, the period of each session was 45-60 minutes. Self-observation accompanied with programmed diary and interaction between patients and therapists were employed in WBT group therapy. The treatment sessions were classified into three phases, initial, intermediate, and final. The first two sessions (the initial phase) were involved in identifying incidences of well-being as well as applying the instructions into situational context regardless of its short period (13). The patients were also asked to give a daily report in which the circumstances of well-being incidences, ranged from 0-100; 0 indicates lack of well-being and 100 illustrates full well-being. In the 3rd to 5th session (the intermediate phase), the patients were encouraged to recognize the thoughts and belief which led to early cutting off of well-being. In the 6th to 8th session (final phase), the patients were assessed by Ryff’s dimensions of PWB scales as follows: i. Environmental mastery, ii. Personal growth, iii. Purpose in life, iv. Autonomy, v. Self-acceptance, and vi. Positive relations with others (18). Therefore, the therapist's objective was to direct patients from a dysfunctional level to an optimal one based on the above-mentioned six dimensions.

During the therapy, the experimental group (n=11) was treated with standard WBT techniques, while the control group (n=11) was on the waiting list for therapy. Therefore, at the end of the sessions, post-assessment was conducted for each client using DASS-21 and PWB.

### Statistical analysis

The obtained data from experiential and control groups were analyzed for normally distributed variables using Levene’s test, and analysis of covariance (ANCOVA) was then utilized to assess the depression scores. Statistical significance was defined by P<0.05.

## Results

Twenty-two infertility women were assigned into the WBT intervention group. Eleven (50%) women considered as experimental group had the mean age of 27.45 (SD=3.62), while the other 11 women (50%) presented as control group had the mean age of 28.18 (SD=4.29). Descriptive statistics and demographic characteristics are presented in Tables 1 and 2.

According to Levene’s test, there are no significant differences regarding the scores of PWB between experimental and control groups, showing the equality of variances (Table 3). Therefore, presumption of ANCOVA for the depression score was established, indicating there are significant differences regarding depression scores between experimental and control groups (P=0.000, Table 4).

**Table 1:** Descriptive statistics for age and duration of infertility


Demographic characteristics		n	Range	Minimum	Maximum	Mean	SD	SE

Experimental group	Age	11	11	23	34	27.45	3.62	1.09
	Duration of infertility	11	4	3	6	4.18	1.27	0.38
Control group	Age	11	15	22	37	28.18	4.29	1.29
	Duration of infertility	11	4	2	6	3.73	1.17	0.35


**Table 2:** Frequencies and percent statistics for age rate and education level


	Age			Education level	Education	
Age rate	Experimental	Control		Experimental	Control

22-26	6(55%)	5(45%)	Diploma	7(64%)	7(64%)
27-31	3(26%)	4(36%)	Advanced diploma	2(18%)	1(9%)
32-37	2(19%)	2(19%)	Bachelor	2(18%)	3(27%)
Sum	11(100%)	11(100%)	Sum	11(100%)	11(100%)


**Table 3:** Data analysis using Levene’s test for equality of variances and ANCOVA for PWB scales


Levene’s test				ANCOVA					
F	df1	df2	Sig	Source Changes	Sum of Squares	df	Mean Squares	F value	P value

0.896	1	20	0.355	Pretest	12758.188	1	12758.188	41.110	0.000
				Group^*^pretest	25.113	1	25.113	0.081	0.779


ANCOVA; Analysis of covariance, F; Function, df; Degree of freedom, Sig; Significant level, *; Correlation is significant at the 0.05 level, and PWB scales; Psychological Well-being scales.

**Table 4:** Data analysis using Levene’s test for equality of variances and ANCOVA for depression scores


Levene’s test				ANCOVA					
F	df1	df2	Sig	Source Changes	Sum of Squares	df	Mean Squares	F value	P value

0.268	1	20	0.610	Pretest	159.381	1	159.381	25.827	0.000
				Group*pretest	0.638	1	0.638	0.103	0.751


ANCOVA; Analysis of covariance, F; Function, df; Degree of freedom, Sig; Significant level, and *; Correlation is significant at the 0.05 level.

**Fig.2:**
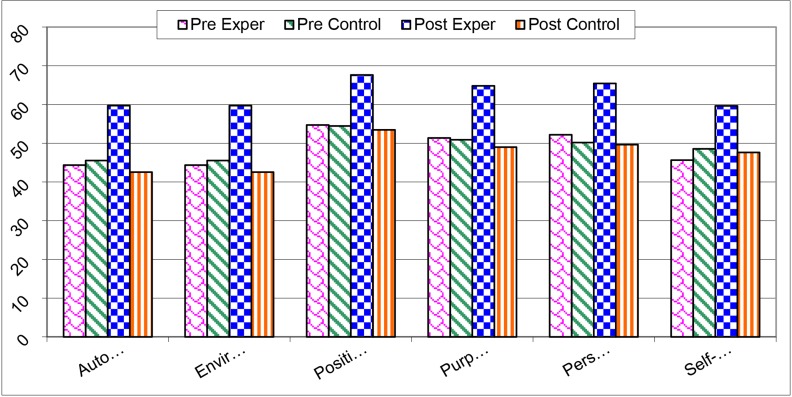
The differences regarding PWB scale preand post-treatment between experimental and control groups. PWB scales; Psychological well-being scales.

The Cohen’s effect size (23) and Cohen’s d values were calculated for preand posttreatment for experimental and control groups (0.646 and 1.693, respectively), suggesting that the experimental group had greater effect than control group. The dimensions of PWB were also subjected to ANCOVA, indicating that the two groups showed significant differences in all dimensions (Table 5, Fig .2).

According to Table 5, it is clearly showed an increase in well-being state causes a decrease in depression, depicted in Table 6, as well.

It indicates that well-being state increased after treatment by WBT (Table 7).

Our findings are in agreement with the previous sources, reviewed in detail elsewhere (24). The present study showed that all infertile women who took part in the eight-session program improved more and showed close relationships, optimistic, happiness, innocence, success, and socialization than the infertile women who did not participate in the program. Therefore, the findings suggested that WBT intervention could be more effective. Furthermore, the correlation between post DASS-21 and post PWB scores was calculated. Also, Pearson correlation revealed (Table 8) that there is a significant negative relationship between (r=-0.601, P=0.003) well-being and depression scores, meaning that when well-being increases after treatment, depression decreases.

**Table 5:** Comparison of the Ryff’s dimensions of PWB scales for pre- and post-treatment between experimental and control groups


Group	Pre exper		Pre control		Post exper		Post control		Significant	
Variable	Mean	SD	Mean	SD	Mean	SD	Mean	SD	F value	P value

Total PWB	297.72	32.64	300.45	27.63	383.45	35.02	289.90	25.79	41.11	0.000
Autonomy	44.36	5.85	45.54	5.69	59.72	7.26	42.54	4.56	16.951	0.001
Environment	44.36	5.85	45.54	5.69	59.72	7.26	42.54	4.56	15.288	0.001
Positive relation	54.72	7.73	54.45	3.41	67.63	7.47	53.45	4.74	6.132	0.023
Purpose in life	51.36	7.71	50.90	6.10	64.81	5.26	49.00	6.58	50.224	0.000
Personal growth	52.18	6.08	50.18	7.99	65.45	5.42	49.63	9.16	44.404	0.000
Self-acceptance	45.63	8.65	48.54	7.98	59.63	8.74	47.63	7.94	100.19	0.000


F; Function, df; Degree of freedom, Sig; Significant level, PWB scales; Psychological Well-being scales.

**Table 6:** Comparison of PWB and DASS-21scales for pre- and post-treatment between experimental and control groups


Groups	n=11		PWB scales		DASS-21 scale	

Experimental			Pre	Post	Pre	Post
		Mean	297.72	383.45	20.90	7.27
		SD	32.64	35.02	1.86	3.60
Control		Mean	300.45	289.90	21.09	23.09
		SD	27.63	25.79	2.87	4.03


All data are presented as mean ± SD. PWB scales; Psychological Well-being scales, and DASS-21 scale; Depression, Anxiety and Stress Scale-21 scale.

**Table 7:** Comparison of the effect size values of PWB and DASS-21scales between two groups


	Experimental group	Control group

Cohen’s d	2.8559257217923335	8.10277696158679
Cohen’s r (effect size)	0.8191171025474095	0.9708625601696644


PWB scales; Psychological Well-being scales, and DASS-21 scale; Depression, Anxiety and Stress Scale-21 scale.

**Table 8:** Comparison of Pearson correlation coefficients between scores of pre-post Dass21 and pre-post PWB scales


	Pre PWB	Post PWB	Pre DASS	Post DASS

Pre well-being Pearson correlation	1	0.497^*^	0.170	-0.217
Sig. (2-tailed)		0.019	0.448	0.332
N	22	22	22	22
Post well-being Pearson correlation	0.497^*^	1	0.066	-0.601^**^
Sig. (2-tailed)	0.019		0.771	0.003
N	22	22	22	22
Pre DASS Pearson correlation	0.170	0.066	1	0.448^*^
Sig. (2-tailed)	0.448	0.771		0.037
N	22	22	22	22
Post DASS Pearson correlation	-0.217	-0.601^**^	0.448^*^	1
Sig. (2-tailed)	0.332	0.003	0.037	
N	22	22	22	22


DASS21; Depression, Anxiety and Stress Scale, PWB scales; Psychological Well-being scales, *; Correlation is significant at the 0.05 level (2-tailed), and **; Correlation is significant at the 0.01 level (2-tailed).

## Discussion

In a study by Fava et al. (25), they have evaluated the efficacy of WBT in patients with recurrent major depression disorder (MDD). Their results indicated that the level and severity of depressive symptoms in MDD patients after treatment with WBT were substantially reduced and a 6-year follow-up was also confirmed this content. In the latest research by Moeenizadeh and Kumar (26), it was pointed out that the application of WBT reduced the symptoms of depression and increased the psychological well-being. A question then arises that why the growth on positive psychology leads to a reduction in depressive symptoms in patients. The answer to this question should be searched in a study that examines the benefits of positive emotions and other feeling. In this regard, it should be noted that positive emotions not only make people feel good about themselves, but also influence the various aspects of a person’s life such as marital satisfaction, interpersonal relationships, career success and good physical conditions (27). Lyubomirsky et al. (28) have shown a positive association between cognitive interventions and reduced symptoms of depression, leading to presence of positive emotions. Therefore, when moderating variables such as positive behavior, positive thoughts and positive emotions are accompanied by positive activities, depressive symptoms are replaced with happiness and feeling of well-being. Such results lead to represent the following model by Layous et al. (8), in which they have indicates the effectiveness of cognitive interventions in reducing depressive symptoms and increasing positive well-being in the clients.

In a meta-analysis of 51 studies, Sin and Lyubomirsky (29) have found that positive psychotherapy interventions were effective prospectively in increasing the well-being and reducing depressive symptoms. In another study by Wood and Joseph (30), they have shown that people earning low scores in PWB scales (regardless of the general structure or one of its constituent dimensions) are at the risk of depression. Their model has revealed that people with lower well-being are depressed seven times more than those with higher well-being in the next 10 years.

In Iran, the relationship between well-being and depression was assessed on 410 women in a study conducted by Ghodsi and Hojjatoleslami (31). The results of this study have revealed that there was no depression among women who had a high sense of well-being. According to their results, an increase in well-being is likely to elicit a reduced risk of depression. The researcher utilized a new method of therapeutic intervention of WBT on women suffering from infertility problems. Their findings are in line with other studies applying WBT to enhance psychological well-being in order to reduce emotional disorders and depression (11, 12).

As in current study, the use of WBT intervention caused to reduce the negative emotions such as depression and increase well-being. According to this treatment, the clients were encouraged to identify their positive emotions in different situations and try to improve them. Our findings revealed that the infertile women in experimental group showed a significant decrease in depression. About the effect sizes, experimental group also showed greater symptom reduction than control group, meaning that there is significant differences in PWB scores. Our results, therefore, indicated that treatment group showed significant improvements in all six dimensions of PWB, confirming the efficacy of this novel technique.

Perhaps the greatest limitation of this study is related to the lack of a follow-up program. Follow-up program could not be conducted because some of infertility women were in sensitive condition of infertility which prevented researchers from contacting them, or there were some limitations coming from their husbands/spouses. Furthermore, we only included the infertile women referred to Montasariya Infertility Center, so our findings cannot be generalized to other population.

## Conclusion

The objective of this study was achieved through helping infertile women to reach optimal level of well-being and realize their true potential. Being pessimistic may be a risk factor for IVF treatment failure. Our results suggested the feasibility and clinical advantages of adding WBT to repertoire of the treatment techniques for depression in infertile women. The future studies are required to explain how pessimism can have a negative effect on results of therapeutic methods through biological and behavioral mechanisms.
